# Acute Generalized Exanthematous Pustulosis: Pathogenesis, Genetic Background, Clinical Variants and Therapy

**DOI:** 10.3390/ijms17081214

**Published:** 2016-07-27

**Authors:** Laurence Feldmeyer, Kristine Heidemeyer, Nikhil Yawalkar

**Affiliations:** Department of Dermatology, Inselspital, Bern University Hospital, University of Bern, Bern 3010, Switzerland; Kristine.Heidemeyer@insel.ch (K.H.); Nikhil.Yawalkar@insel.ch (N.Y.)

**Keywords:** acute generalized exanthematous pustulosis, dermatology, skin, drug reaction

## Abstract

Acute generalized exanthematous pustulosis (AGEP) is a severe, usually drug-related reaction, characterized by an acute onset of mainly small non-follicular pustules on an erythematous base and spontaneous resolution usually within two weeks. Systemic involvement occurs in about 20% of cases. The course is mostly benign, and only in rare cases complications lead to life-threatening situations. Recent studies highlight the importance of genetic variations in interleukin-36 receptor antagonist gene (*IL-36RN*) in the pathogenesis of this disease. The physiopathology of AGEP remains unclear, but an involvement of innate and acquired immune cells together with resident cells (keratinocytes), which recruit and activate neutrophils via production of cytokines/chemokines such as IL-17, IL-36, granulocyte-macrophage colony-stimulating factor (GM-CSF), tumor necrosis factor alpha (TNFα) and chemokine (C-X-C motif) ligand 8 (CXCL8)/IL-8, has been postulated. Treatment is based on the removal of the causative drug, supportive care, infection prevention and use of potent topical or systemic steroids.

## 1. Introduction

Cutaneous adverse reactions to drugs are common and encompass a variety of mild to severe and life-threatening reactions. Acute generalized exanthematous pustulosis (AGEP) represents a severe, usually drug-related skin reaction characterized by acute formation of sterile pustules on an erythematous background, fever and neutrophilia. 

## 2. Background and Epidemiology

While the clinical picture of drug-induced pustular eruptions in patients without any history of psoriasis had already been described in 1968 by Baker and Ryan, the term AGEP was introduced by Beylot et al. in 1980 [1,2]. AGEP is a rare adverse drug reaction with an incidence of one to five cases per million per year [3], but it might be underreported. It can occur at any age and seems to be more frequent in women [4].

## 3. Aetiology

Although many causative factors leading to AGEP have been described, it is, in over 90% of cases, associated with the ingestion of drugs [5,6]. Aminopenicillins, pristinamycin, sulphonamides, quinolones, hydroxychloroquine, terbinafin and diltiazem are the most frequent causative drugs [7]. In particular cases, AGEP is induced by bacterial, viral or parasitic infections (e.g., parvovirus B19 [8,9], mycoplasma [10,11], cytomegalovirus [12], coxsackie B4 [13], *Chlamydia pneumoniae* [14], *Escherichia coli* [15], and echinococcus [16]), spider bites [17], herbal medications [18], lacquer [18], mercury [19] and even psoralen combined with ultraviolet A (PUVA) treatment [20]. Finally venoms, foods and xenobiotics have also been suspected to induce the reaction [21].

## 4. Genetic Background

The genetic predisposition for the development of AGEP is not known. There seems to be a correlation between mutations in the *IL-36RN* gene, encoding the interleukine-36 receptor antagonist (IL-36Ra), and the development of generalized pustular eruptions after drug intake. IL-36Ra has an anti-inflammatory function and blocks the proinflammatory cytokines IL-36α, IL-36β and IL-36γ. Mutations in the *IL-36RN* gene can result in uncontrolled IL-36 signalling and increased downstream production of further proinflammatory cytokines and chemokines [22]. However, it is still unclear if mutations in *IL-36RN* lead to AGEP or, rather, to a drug-induced generalized pustular psoriasis (GPP), as it is described in some cases [23,24].

## 5. Pathogenesis

**Figure 1:**
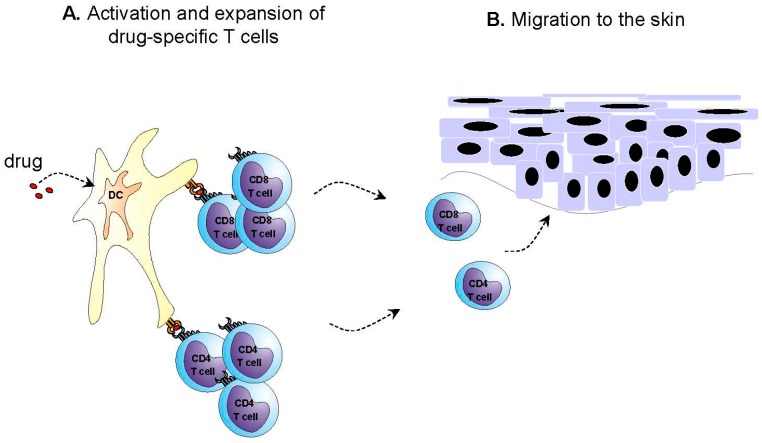

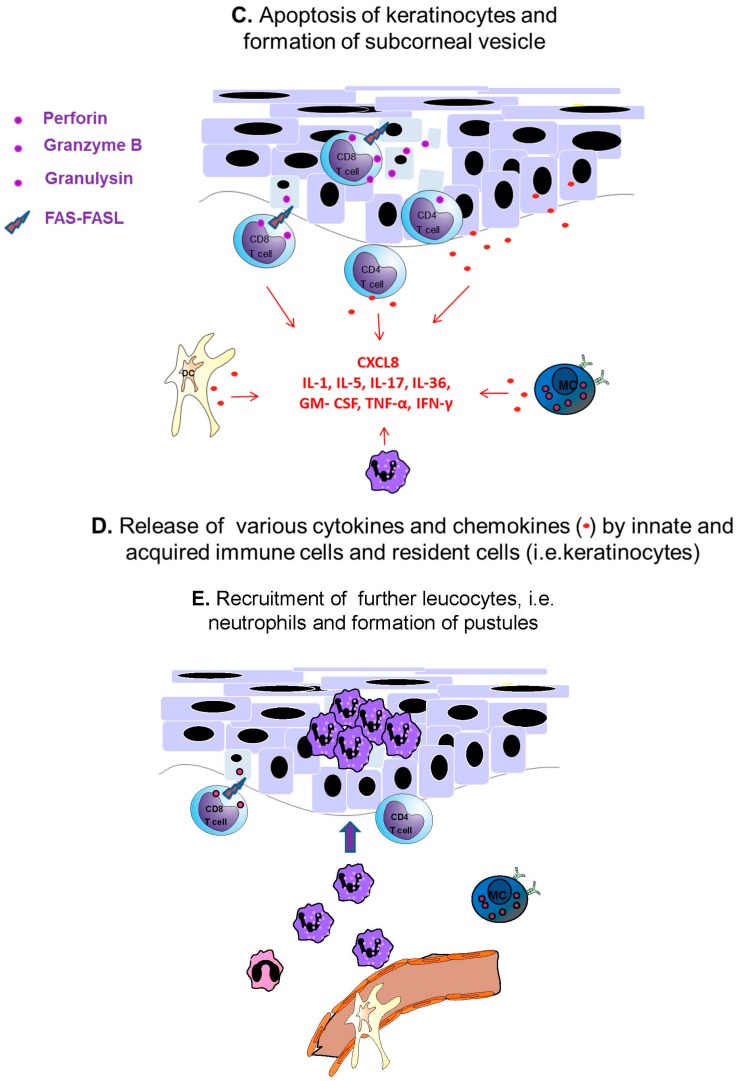
Putative pathogenic mechanisms in acute generalized exanthematous pustulosis (AGEP). (**A**) In cases with drug involvement, the initial phase involves stimulation of drug-specific T cells and (**B**) their migration to the skin; (**C**) These T cells, possibly together with natural killer T (NKT) cells/natural killer (NK) cells are activated in the skin, where they induce apoptosis of keratinocytes through cytotoxic proteins and Fas/Fas ligand (FasL) interactions resulting in the formation of subcorneal vesicles; (**D**) Furthermore, these T cells together with subsequently activated bystander and inflammatory cells (keratinocytes, dendritic cells (DC), MC, neutrophils) release various cytokines and chemokines; (**E**) which predominately lead to neutrophilic inflammation and the formation of pustules.

AGEP has been classified as a T cell-related sterile neutrophilic inflammatory response (type IVd reaction) [25,26,27]. The activation, proliferation and migration of drug-specific cluster of differentiation (CD) 4 and CD8 T cells play an important role in the development of AGEP (Figure 1), as supported by the use of patch tests [17,18,19,20] and in vitro tests [21,22]. It is supposed that drug-specific cytotoxic T cells and cytotoxic proteins such as granzyme B and perforin induce the apoptosis of keratinocytes, leading to subcorneal vesicles [27,28]. Recently, it has also been shown that, besides in toxic epidermal necrolysis (TEN), granulysin is also expressed by CD4 and CD8 T cells and natural killer (NK) cells in different drug reactions including AGEP, suggesting that granulysin may also play a role in the pathogenesis of AGEP [28]. Furthermore, in vitro tests have shown that drug-specific T cells in AGEP patients produced significantly more chemokine (C-X-C motif) ligand 8 (CXCL8)/IL-8, a potent neutrophil chemotactic chemokine [26,27,28,29]. CXCL8/IL-8 is thought to play a central role in the formation of pustules by recruitment of neutrophils. The increased levels of IL-17 and IL-22 as well as granulocyte-macrophage colony-stimulating factor (GM-CSF) in AGEP patients may also participate in the strong neutrophilic activity by the synergistic effect on the production of CXCL8/IL-8 and the prevention of apoptosis of the neutrophils [28,29]. Recent studies also described a higher level of IL-17 expression by neutrophils, mast cells (MC), and macrophages, and a lower level by T cells, in AGEP patients, indicating that innate cells may also be involved in the pathogenesis of AGEP [29]. Furthermore, a deficiency in the IL36-Ra in some AGEP patients seems to play a role, leading to the increased expression of various proinflammatory cytokines and chemokines such as IL-1, IL-6, IL-12, IL-23, IL-17, tumor necrosis factor alpha (TNFα) and CXCL8/IL-8, which can further enhance neutrophilic recruitment and activation [22,23,30].

In some AGEP patients, IL-5 expressed by infiltrating T cells may lead to the eosinophilia presented in about 30% of AGEP patients [26]. An elevated expression of TNFα in AGEP patients has been reported [31].

## 6. Clinical Features and Variants

**Figure 2:**
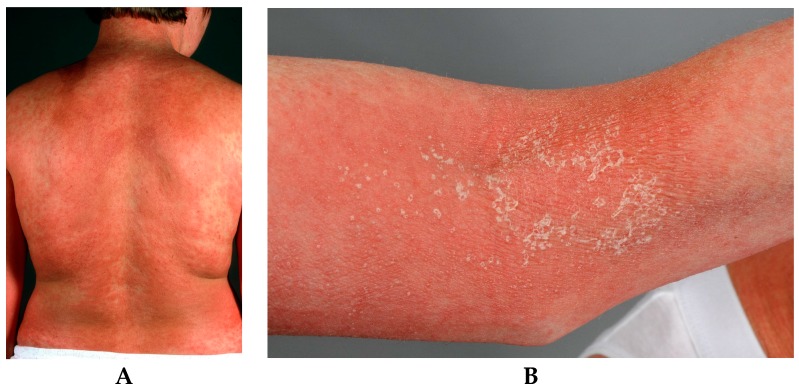
(**A**) Widespread rash with numerous pinhead-sized pustules on an erythematous oedematous base; (**B**) Flexural accentuation with characteristic collarette-shaped desquamation is typically observed in AGEP.

Characteristically, patients with AGEP develop an acute rash with pinhead-sized pustules on an erythematous oedematous base, starting in the main folds (axillary, inguinal and submammary areas) and spreading quickly (within a few hours) on the trunk and limbs (Figure 2). The time period from drug ingestion to reaction onset is usually within 48 h, with antibiotics having a median of 24 h [7]. There is an itching or sometimes burning sensation [3,32]. Mucosal involvement, especially orally, is reported in about 20%–25% of patients but mostly in a limited extension and only on one mucosal region [5].

Systemic inflammation signs in the acute phase of the disease include fever (>38.0 °C), leucocytosis (>10,000/mL), elevated levels of C-reactive protein (CRP) and mostly increased levels of neutrophils (>7000/mL). As mentioned above, 30% of patients also present an eosinophilia and in 75% of cases a hypocalcaemia, probably related to hypoalbuminemia, is found [5,33]. Multiorgan involvement has been reported in 17% of cases [33]. Skin eruptions are sometimes accompanied by lymphadenopathy and occasionally by hepatocellular dysfunction and cholestasis as well as nephritis. Lung and bone marrow might also be involved, leading to respiratory failure and neutropenia, respectively [33]. One case with phenytoin-induced AGEP and cerebellar symptoms has been reported, while it is unknown whether cerebellar symptoms were related to the drug reaction or to phenytoin toxicity [34]. AGEP usually shows a mild course but high fever or cutaneous superinfection can complicate the process and lead to severe illness and sometimes life-threatening situations, especially in patients of poor general condition. The reported mortality is under 5% [3,4]. Usually there is a spontaneous resolution of skin lesions within two weeks with a very typical collarette-shaped desquamation in the region of previous pustulosis [3,4].

Besides the normal presentation of AGEP, several atypical variants and overlap syndromes have been described. For example, an overlap of AGEP and drug reaction with eosinophilia and systemic symptoms (DRESS) [35,36], or TEN [36,37,38,39,40,41,42,43,44], as well as an AGEP case with targetoid lesions [45,46] have been reported. A dozen of localized reactions have been reported, and referred to as acute localized exanthematous pustulosis (ALEP) [47,48,49,50]. In about 50% of patients, additional skin symptoms such as erythematous oedema of the hand and face, purpura, vesicles and blisters have been reported. 

Differential diagnosis of other pustular eruptions (such as bacterial or fungal infections, neutrophilic dermatoses, etc.) can mostly be excluded easily by clinical picture, history and histopathological findings. Acute GPP can present with the same clinical picture and may be difficult to distinguish, as the histopathological findings can sometimes not clearly distinguish between these two diseases (Table 1). The most important factor for the diagnosis is the quicker resolution time seen in AGEP. Recent studies described similarities in the pathogenesis of AGEP and GPP, like mutations in the IL-36Ra and an elevated expression of IL-17 [51]. DRESS typically presents with a morbilliform rash spreading from the face to the rest of the body, but might present with pustules as well. DRESS develops with a longer latent period of two to six weeks and mucous membranes and systemic involvement are more common [52]. Stevens-Johnson syndrome (SJS) and TEN are characterized by epidermal detachment and histological full-thickness epidermal necrosis [53]. Severe cases of AGEP, especially those with mucosal involvement, might be difficult to distinguish from these entities, and overlap forms have been described.

## 7. Histopathological Findings

**Figure 3:**
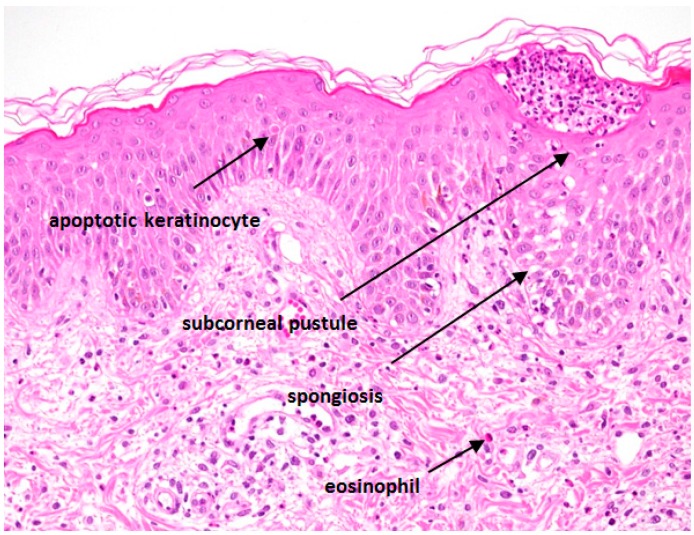
Typical histological features of AGEP are indicated.

A histopathological examination should be performed to distinguish AGEP from other pustular eruptions. The skin biopsy should include a pustule. Typically, the biopsy shows spongiform subcorneal and/or intraepithelial pustules, an oedematous papillary dermis and perivascular infiltrates with neutrophils and some eosinophils (Figure 3). In some cases, necrotic keratinocytes and leucocytoclastic vasculitis can also be found. Histopathologically, it can be difficult to differentiate AGEP from GPP. The presence of eosinophils, necrotic keratinocytes, a mixed interstitial and mid-dermal perivascular infiltrate and absence of tortuous or dilated blood vessels favors AGEP, while the presence of psoriasiform acanthosis is characteristic of GPP [24,54]. 

## 8. Diagnosis

Diagnosis of AGEP can be made clinically with the support of histopathological findings as well as patch tests. The EuroSCAR study group presented a standardized scoring system in 2001, the AGEP validation score, including the morphology of skin lesions, the presence of fever, the clinical course, and the laboratory and histopathological findings [4]. To identify the responsible drug in case of polymedication, a patch test can be performed after complete skin resolution. The sensibility of the patch test in AGEP is higher than in other drug reactions such as SJS or TEN (58% positive in AGEP vs. 24% positive in SJS/TEN). A positive result often shows small pustules at the location of testing [55,56,57].

## 9. Therapy

Discontinuation of the causative agent is the main objective. Due to the mostly benign and self-limiting course, a supportive treatment based on topical steroids and disinfectant solutions during the pustular phase and rehydrating lotions during the desquamative phase, as well as antipyretics, is usually sufficient. In very extensive rashes, the intake of systemic steroids for a short time can be discussed [4], although there is no evidence that they reduce disease duration: their use is empirical and not supported by any randomized study. In one report, there was no difference between different treatment regimens regarding the course and duration of the disease or the length of fever [6,18,58]. Two cases of steroid-induced AGEP have been reported [58].

## 10. Conclusions

Recent research has improved our understanding of the pathophysiology of the disease but so far no markers have been identified that can predict which patients will develop the disease. The establishment of precise diagnostic criteria in AGEP is definitely a fundamental basis for clinical trials of quality [4]. There are currently no published randomized trials for a topical or systemic therapy for AGEP. Such trials are difficult to perform in a rare disease but necessary to provide a definitive answer. It is important to sensitize dermatologists and internists to this diagnosis as we feel that this disease is underreported. Indeed, the rash is usually not life-threatening and has a high rate of spontaneous resolution, so patients are not always referred to a dermatologist.

## Figures and Tables

**Table 1:** Factors favoring the diagnosis of AGEP over pustular psoriasis.

	AGEP	Generalized Pustular Psoriasis
**History of psoriasis (family/personal) **	usually lacking	often present
**Distribution pattern**	initially predominance in the folds	more generalized
**Onset of pustules**	fast (hours or few days after use of medication)	slower
**Duration of pustules**	Shorter (rapid resolution in a few days, max. 15 days, after drug suspension)	longer
**Size of pustules**	tiny (pinhead)	larger
**Duration of eruption/fever**	shorter (resolution in a few days after drug suspension)	longer
**History of drug reaction**	usual	uncommon
**Recent drug administration**	very frequent	less frequent
**Arthritis **	rare	about 30%
**Histology**	single-cell necrosis of keratinocytes, edema of papillary dermis, vasculitis, exocytosis of eosinophils	papillomatosis, acanthosis, tortuous or dilated vessels
